# Patient Retreat in Dose Escalation for Phase I Clinical Trials With Rare Diseases

**DOI:** 10.1002/sim.70409

**Published:** 2026-02-05

**Authors:** Jialu Fang, Guosheng Yin

**Affiliations:** ^1^ Department of Statistics and Actuarial Science The University of Hong Kong Hong Kong China

**Keywords:** Bayesian design, calibration‐free odds design, dose‐finding trial, phase I trial, small sample size

## Abstract

Phase I clinical trials aim to identify the maximum tolerated dose (MTD), a task that becomes challenging in rare disease due to limited patient recruitment. Traditional dose‐finding designs, which assign one dose per patient, require a sufficient sample size that may be infeasible for rare disease trials. To address these limitations, we propose the patient retreat in dose escalation (PRIDE) scheme, which integrates intra‐patient dose escalation and considers intra‐patient correlations by incorporating random effects into a Bayesian hierarchical framework. We further introduce PRIDE‐FA (flexible allocation), an extension of PRIDE with a flexible allocation strategy. By allowing retreated patients to be assigned to any dose level based on trial needs, PRIDE‐FA improves resource efficiency, leading to greater reductions in required sample size and trial duration. This paper incorporates random effects into established dose‐finding designs, including the calibration‐free odds (CFO) design, the Bayesian optimal interval (BOIN) design, and the continual reassessment method (CRM) to account for intra‐patient correlations when each patient may receive multiple doses. Simulation studies demonstrate that PRIDE and PRIDE‐FA significantly improve the accuracy of MTD selection, reduce required sample size, and shorten trial duration compared to existing dose‐finding methods. Together, PRIDE and PRIDE‐FA provide a robust and efficient framework for phase I clinical trials with rare diseases.

## 1 The Bottle Neck of Phase I Trials With Rare Diseases

In phase I cancer clinical trials, the primary objective is to identify the maximum tolerated dose (MTD), defined as the dose at which the probability of dose‐limiting toxicity (DLT) is closest to a pre‐specified target toxicity rate. Trials typically investigate a series of increasing dose levels, assuming that the probability of toxicity and drug efficacy rise with dose [1]. Determining the MTD through the clinical data and statistical modeling is essential to maximize the therapeutic effect of the drug while avoiding unacceptable toxicity.

Trial designs for finding the MTD fall into algorithm‐based, model‐based, and model‐assisted approaches [2]. Algorithm‐based designs, such as the 3+3 design [3], rely on simple, prespecified rules for dose escalation or de‐escalation and are easy to implement but suffer from low accuracy in identifying the MTD. Model‐based designs, such as the continual reassessment method (CRM) [4] and the escalation with overdose control design [5], use statistical models to estimate the dose–toxicity relationship and adaptively guide dose decisions. They offer superior performance but require complex calculations and specialized expertise. Model‐assisted designs combine the simplicity of algorithm‐based designs with the statistical efficiency of model‐based methods. Designs such as the Bayesian optimal interval (BOIN) design [6] and the modified toxicity probability interval design [7] use pre‐tabulated decision rules for straightforward implementation while maintaining good performance. However, these designs only focus on the current dose level when making decisions, ignoring the information from other doses. The calibration‐free odds (CFO) design [8], a Bayesian model‐assisted framework, avoids explicit dose–toxicity modeling and leverages data across multiple doses for decision making.

Despite these advances, most existing dose‐escalation methods are not well‐suited for rare disease trials, where patient recruitment is constrained by low prevalence, heterogeneous clinical presentations, and small eligible populations. Phase I trials typically range from 30 to 50 participants, but even this modest target is often difficult to meet within a reasonable trial period. For instance, traditional early‐phase trials in Duchenne muscular dystrophy (DMD) face operational barriers due to the disease's rapid progression and small eligible populations [9]. An illustrative example is a phase I/II trial of viltolarsen in Japanese patients that enrolled only 16 participants with DMD [10]. Spinal muscular atrophy presents similar issues, including a narrow recruitment window and the limited number of eligible patients [11], as seen in the phase I trial of onasemnogene abeparvovec with only 13 patients enrolled [12]. In fibrodysplasia ossificans progressiva (FOP), the extreme rarity and high inter‐patient variability in treatment response make conventional trial designs impractical [13]. A phase I trial investigated palovarotene in FOP and enrolled only 20 participants [14].

Existing dose‐finding methods assume that each patient receives only one dose, requiring a large sample size to explore the dose range and determine the MTD. This single‐patient, single‐dose mechanism is inefficient for small‐sample trials and may expose patients to subtherapeutic doses. Intra‐patient dose escalation (IPDE) addresses these limitations by allowing patients to receive progressively higher doses, generating more data per patient and enhancing dose exploration [15]. However, traditional IPDE designs, such as Simon's accelerated titration design [15], often rely solely on individual patient data and lack information sharing across dose levels, increasing the risk of unreliable estimation. Innovative dose‐escalation designs have been developed to address these limitations. Xu et al. [16] developed a design for T‐cell engagers that combines intra‐patient dose escalation with survival analysis to estimate the MTD. Zhou et al. [17] introduced the adaptive intra‐patient dose escalation (AIDE) strategy, which uses both individual and cohort‐level toxicity data to guide dose escalation, reducing the required sample size while ensuring patient safety.

In the aforementioned dose‐escalation methods, a patient receiving multiple doses across treatment cycles is treated as a “new patient” at each dose level. Toxicity outcomes for one patient at different dose levels are assumed to be independent. In reality, patient‐specific factors, such as physiological characteristics, drug metabolism, disease progression, and cumulative treatment effects, introduce intra‐patient correlations across dose levels. Ignoring these correlations may result in biased toxicity probability estimates, leading to suboptimal dose movement decisions. Guo and Liu [18] recently introduced the intra‐patient CRM with random effects, which incorporates random effects into the CRM design. To systematically explore how random effects can be incorporated into a dose‐finding trial, we propose the patient retreat in dose escalation (PRIDE) scheme, which accounts for intra‐patient correlations by introducing random effects into a hierarchical logistic regression model. To further enhance sample size efficiency and shorten trial duration, we introduce PRIDE‐FA (flexible allocation), an extension of the PRIDE framework that allows retreated patients to be reassigned flexibly based on trial needs. Among dose‐finding designs, the CFO design has been shown to perform optimally in most scenarios for Phase I clinical trials [8, 19, 20, 21]. Thus, the simulations and trial illustrations focus on applying PRIDE and PRIDE‐FA to the CFO design.

The remainder of this paper is organized as follows: Section 2 introduces the PRIDE and PRIDE‐FA schemes, and their implementation in CFO, BOIN, and CRM designs. Section 3 presents simulation experiments conducted under both fixed and random scenarios. Section 4 illustrates the application of PRIDE and PRIDE‐FA through a trial example using the CFO design. We conclude with a brief discussion in Section 5.

## 2 Methods for Addressing Shortage of Samples

### 2.1 PRIDE With Random Effects

Consider a trial investigating K dose levels with increasing DLT rates, $p_{1}<\ldots <p_{K}$, and a pre‐specified target DLT rate ϕ. After each cohort of patients is enrolled, the next dose level is assigned based on observed toxicity outcomes from previous cohorts. Traditional phase I dose‐finding methods assign each patient to a single dose, contributing only one observation to evaluate the dose–toxicity relationship of the drug. This inter‐patient dose escalation strategies limit the ability of the trial design to identify the MTD with minimal sample size. For instance, with five dose levels under investigation and 12 recruited patients, if each cohort consists of three patients and dose skipping is prohibited, the design even fails to test all dose levels due to exhaustion of the total sample size up to dose level 4.

To make efficient use of enrolled patients, Zhou et al. [17] proposed the AIDE design, enabling intra‐patient dose escalation by retreating patients who have not experienced DLTs. Instead of assigning a new cohort to the next dose level ($d_{\text{next}}$), AIDE retreats previously enrolled patients who have tolerated all $d_{\text{next}}-1$ lower dose levels without experiencing DLT. Cohorts at $d_{\text{next}}$ may thus include both “retreated” and newly enrolled patients. This approach reduces sample size, requiring new enrollment only when retreated patients are insufficient. However, the same patient may receive multiple doses, introducing intra‐patient correlation. This issue is particularly critical for dose‐finding designs such as the CRM and CFO designs (both use observed data at multiple doses for decision making), as multiple‐dose observations contributed by the same patient would influence dose decisions. To address this, we propose the PRIDE scheme, which accounts for intra‐patient correlation by incorporating random effects into the model. In PRIDE, retreated patients are assigned to higher doses during dose escalation, and a washout period is applied to mitigate carryover effects from previous dosage treatment.

During the trial, suppose that n patients have been enrolled, with the observed toxicity outcomes as $D_{n}$. Let $p_{\mathrm{ik}}$ be the probability of toxicity and $y_{\mathrm{ik}}$ the DLT outcome for patient i at dose level k, with i=1,…,n and k=1,…,K. This setting resembles a repeated measurement study, where each subject may be measured multiple times longitudinally, resulting in correlated outcomes within a subject. Under the PRIDE scheme, a patient either experiences a DLT at his/her initially assigned dose level (in which case they do not undergo retreatment) or no DLT is observed, thus allowing the patient to be retreated. Once a DLT occurs at a specific dose level, the retreatment scheme for that particular patient stops. Each patient is allowed to be treated at up to three different dose levels, with each retreatment occurring only at a higher dose than the previous one. The observed DLT vector consists of a sequence of 0 corresponding to the absence of DLT at each dose level, followed by the last value being either 0 or 1, where 0 indicates that the patient is still eligible for retreatment, and 1 indicates that the patient will no longer be retreated due to having experienced the DLT.

To account for intra‐patient correlations arising from multiple observations per patient, we employ a Bayesian hierarchical model. The DLT rate for patient i at dose level k, denoted as $p_{\mathrm{ik}}$, is modeled using a logistic regression model as

$$p_{\mathrm{ik}}=\frac{\exp\left(\beta_{k}+W_{i}\right)}{1+\exp\left(\beta_{k}+W_{i}\right)},\;i=1,\ldots ,n;\;k=1,\ldots ,K,$$

where $\beta_{k}$ is the dose‐specific fixed effect, describing the overall dose–toxicity trend, and $W_{i}$ is the patient‐specific random effect, capturing individual deviations from this trend. The inclusion of $W_{i}$ links observed data at different doses from the same patient, thus modeling intra‐patient correlations.

Observations for certain combinations of patients and dose levels may be missing in dose‐finding trials. Specifically, if a patient experiences the DLT at dose level k, no further observations will be recorded at higher dose levels for that patient, as the patient with DLT is no longer eligible to be retreated. To formalize this, we define an indicator function $I_{\mathrm{ik}}$, where $I_{\mathrm{ik}}=1$ if the DLT outcome $y_{\mathrm{ik}}$ exists (patient i has been treated at dose level k), and $I_{\mathrm{ik}}=0$ otherwise. Using this indicator, the conditional likelihood given the observed data $D_{n}$ is expressed as

$$\begin{array}{cc} L\left(\beta |\;W,D_{n}\right) & =\prod_{i=1}^{n}\prod_{k=1}^{K}{\left\{p_{\mathrm{ik}}^{y_{\mathrm{ik}}}{\left(1-p_{\mathrm{ik}}\right)}^{1-y_{\mathrm{ik}}}\right\}}^{I_{\mathrm{ik}}}\\ & \;=\prod_{i=1}^{n}\prod_{k=1}^{K}{\left\{\frac{\exp\left(\beta_{k}+W_{i}\right)}{1+\exp\left(\beta_{k}+W_{i}\right)}\right\}}^{y_{\mathrm{ik}}I_{\mathrm{ik}}}{\left\{\frac{\exp\left(\beta_{k}+W_{i}\right)}{1+\exp\left(\beta_{k}+W_{i}\right)}\right\}}^{\left(1-y_{\mathrm{ik}}\right)I_{\mathrm{ik}}}, \end{array}$$

where $\beta ={\left(\beta_{1},\ldots ,\beta_{K}\right)}^{⊤}$ represents the fixed effects and $\;W={\left(W_{1},\ldots ,W_{n}\right)}^{⊤}$ represents the random effects.

Under the Bayesian hierarchical model, prior distributions are specified to enable posterior inference. For the fixed effects, we assume $\beta_{k}∼𝒩\left(\mu_{k},\sigma_{\beta }^{2}\right)$, where $\sigma_{\beta }^{2}$ controls the prior uncertainty around the mean $\mu_{k}$. To ensure a monotonic dose–toxicity relationship (i.e., $p_{\mathrm{ik}}$ increases with k), we impose the constraint $\mu_{1}<\ldots <\mu_{K}$, with $\mu_{k}=\text{logit}\left(c_{k}\right)$, where $\left\{c_{k}\right\}$ is a pre‐specified increasing sequence of the expected toxicity probabilities (e.g., $c_{k}\in \left\{0.1,\ldots ,0.5\right\}$ for a five‐dose trial). For the random effects, we assume $W_{i}|\sigma^{2}∼𝒩\left(0,\sigma^{2}\right)$, with $\sigma^{2}∼\mathrm{Inv}‐\text{Gamma}\left(\eta ,\eta \right)$, where η controls the prior uncertainty about $\sigma^{2}$. Setting η=1 represents a common choice for a non‐informative prior. Given these priors and the observed data $D_{n}$, the joint posterior distribution is

$$\begin{array}{cc} & p\left(\beta ,\;W,\sigma^{2}|D_{n}\right)\propto f\left(\sigma^{2}\right)\prod_{i=1}^{n}\prod_{k=1}^{K}{\left\{\frac{\exp\left(\beta_{k}+W_{i}\right)}{1+\exp\left(\beta_{k}+W_{i}\right)}\right\}}^{y_{\mathrm{ik}}I_{\mathrm{ik}}}\\ & \;{\left\{\frac{1}{1+\exp\left(\beta_{k}+W_{i}\right)}\right\}}^{\left(1-y_{\mathrm{ik}}\right)I_{\mathrm{ik}}}f\left(W_{i}\right)f\left(\beta_{k}\right), \end{array}$$

where $f\left(\sigma^{2}\right)$, $f\left(W_{i}\right)$, and $f\left(\beta_{k}\right)$ denote the prior distributions for $\sigma^{2}$, $W_{i}$, and $\beta_{k}$, respectively.

To enable implementation of a Gibbs sampler, we derive the full conditional distributions of the parameters. For i=1,…,n and k=1,…,K, the full conditional distributions (with $\beta_{k}$ and $W_{i}$ expressed in logarithmic forms) are

$$\begin{array}{cc} & \;\log p\left(\beta_{k}|\;W,D_{n}\right)\propto \sum_{i=1}^{n}I_{\mathrm{ik}}\left[y_{\mathrm{ik}}\left(\beta_{k}+W_{i}\right)-\log\left\{1+\exp\left(\beta_{k}+W_{i}\right)\right\}\right]-\frac{{\left(\beta_{k}-\mu_{k}\right)}^{2}}{2\sigma_{\beta }^{2}},\\ & \log p\left(W_{i}|\beta ,\sigma^{2},D_{n}\right)\propto \sum_{k=1}^{K}I_{\mathrm{ik}}\left[y_{\mathrm{ik}}\left(\beta_{k}+W_{i}\right)-\log\left\{1+\exp\left(\beta_{k}+W_{i}\right)\right\}\right]-\frac{W_{i}^{2}}{2\sigma^{2}},\\ & \;p\left(\sigma^{2}|\;W,D_{n}\right)\propto {\left(\sigma^{2}\right)}^{-\left(\eta +1+\frac{n}{2}\right)}\exp\left(-\frac{\eta }{\sigma^{2}}-\frac{\sum_{i=1}^{n}W_{i}^{2}}{2\sigma^{2}}\right). \end{array}$$

Due to the conjugate prior structure, the posterior distribution of $\sigma^{2}$ is

$$\sigma^{2}|W,D_{n}∼\mathrm{Inv}‐\text{Gamma}\left(\eta +\frac{n}{2},\eta +\frac{\sum_{i=1}^{n}W_{i}^{2}}{2}\right).$$

We use the Metropolis–Hastings algorithm within Gibbs sampling to iteratively sample from the full conditional distributions and approximate the joint posterior. Posterior samples are then used to estimate quantities of interest.

### 2.2 PRIDE‐FA: PRIDE With Flexible Allocation

PRIDE enables efficient dose escalation by retreating patients and modeling intra‐patient correlations. However, it is less flexible when certain dose levels lack sufficient data or when adaptive allocation is desired. To address this, we introduce PRIDE‐FA, an extension of the PRIDE framework that employs a more flexible allocation (FA) strategy. PRIDE‐FA allows retreated patients to be assigned to any dose level (including lower doses), as long as that dose has not been previously administered to the same patient. To ensure that at each dose all the observations are from different patients, PRIDE‐FA does not reassign a patient to the previously received doses by the same patient.

Patients who have tolerated higher doses without experiencing DLTs may have greater drug tolerance. Thus, those reassigned to lower doses after tolerating higher doses may show different intra‐individual correlations compared to those reassigned to higher doses. Newly enrolled patients and those retreated at higher dose levels are similar, because they both face higher and potentially riskier doses with unknown tolerance. Therefore, it is reasonable to assume that the correlation structure among these two groups is comparable. We categorize each patient–dose assignment using a binary indicator $Z_{\mathrm{ik}}\in \left\{1,2\right\}$, where $Z_{\mathrm{ik}}=1$ indicates that patient i is either newly enrolled or up‐retreated at dose level k, and $Z_{\mathrm{ik}}=2$ indicates a down‐retreated patient.

To capture the heterogeneity between two assignment types, we introduce a scaling parameter $\alpha_{Z_{\mathrm{ik}}}$. The patient‐specific random effect $W_{i}∼𝒩\left(0,\sigma^{2}\right)$ is modulated by $\alpha_{Z_{\mathrm{ik}}}$, such that the model for patient i at dose k is

$$\text{logit}\left(p_{\mathrm{ik}}\right)=\beta_{k}+\alpha_{Z_{\mathrm{ik}}}W_{i}.$$

We fix $\alpha_{1}=1$ for newly enrolled and up‐retreated patients, and take $\alpha_{2}$ as unknown for down‐retreated patients. The priors for $\beta_{k}$, $W_{i}$, and $\sigma^{2}$ remain as previously described. For $\alpha_{2}$, a normal prior $𝒩\left(1,\sigma_{\alpha }^{2}\right)$ is adopted. The joint posterior distribution is

$$\begin{array}{cc} & p\left(\beta ,\;W,\sigma^{2},\alpha_{2}|D_{n}\right)\propto f\left(\sigma^{2}\right)f\left(\alpha_{2}\right)\prod_{i=1}^{n}\prod_{k=1}^{K}{\left\{\frac{\exp\left(\beta_{k}+\alpha_{Z_{\mathrm{ik}}}W_{i}\right)}{1+\exp\left(\beta_{k}+\alpha_{Z_{\mathrm{ik}}}W_{i}\right)}\right\}}^{y_{\mathrm{ik}}I_{\mathrm{ik}}}\\ & \;{\left\{\frac{1}{1+\exp\left(\beta_{k}+\alpha_{Z_{\mathrm{ik}}}W_{i}\right)}\right\}}^{\left(1-y_{\mathrm{ik}}\right)I_{\mathrm{ik}}}f\left(W_{i}\right)f\left(\beta_{k}\right). \end{array}$$

We derive the log‐form full conditional distributions for the parameters $\beta_{k}$, $W_{i}$, and $\alpha_{2}$, for each patient i and dose level k, as follows,

$$\begin{array}{cc} & \;\log p\left(\beta_{k}|\;W,\alpha_{2},D_{n}\right)\propto \sum_{i=1}^{n}\;I_{\mathrm{ik}}\left[y_{\mathrm{ik}}\left(\beta_{k}+\alpha_{Z_{\mathrm{ik}}}W_{i}\right)-\log\left\{1+\exp\left(\beta_{k}+\alpha_{Z_{\mathrm{ik}}}W_{i}\right)\right\}\right]\\ & \;-\frac{{\left(\beta_{k}-\mu_{k}\right)}^{2}}{2\sigma_{\beta }^{2}},\\ & \log p\left(W_{i}|\beta ,\sigma^{2},\alpha_{2},D_{n}\right)\propto \sum_{k=1}^{K}\;I_{\mathrm{ik}}\left[y_{\mathrm{ik}}\left(\beta_{k}+\alpha_{Z_{\mathrm{ik}}}W_{i}\right)-\log\left\{1+\exp\left(\beta_{k}+\alpha_{Z_{\mathrm{ik}}}W_{i}\right)\right\}\right]\\ & \;-\frac{W_{i}^{2}}{2\sigma^{2}},\\ & \;\log p\left(\alpha_{2}|\beta ,\;W,D_{n}\right)\propto \sum_{i=1}^{n}\sum_{k=1}^{K}\;I_{\mathrm{ik}}\cdot I\left(Z_{\mathrm{ik}}=2\right)\left[y_{\mathrm{ik}}\left(\beta_{k}+\alpha_{2}W_{i}\right)\right.\\ & \;\left.-\log\left\{1+\exp\left(\beta_{k}+\alpha_{2}W_{i}\right)\right\}\right]-\frac{{\left(\alpha_{2}-1\right)}^{2}}{2\sigma_{\alpha }^{2}}. \end{array}$$

The full conditional distribution of $\sigma^{2}$ remains identical to that in PRIDE.

### 2.3 Implementation of the PRIDE Scheme in CFO

We integrate the PRIDE scheme into the CFO design [8] to demonstrate its implementation in dose‐finding trials. The CFO design has been shown to outperform other popular methods, offering a robust, model‐free, and calibration‐free framework, making it well‐suited for incorporating PRIDE. Given K doses and n enrolled patients, observed toxicity outcomes at all dose levels are $D_{n}={\left\{y_{k},n_{k}\right\}}_{k=1}^{K}$, where $y_{k}$ and $n_{k}$ represent the number of observed DLTs and the number of patients treated at dose level k, respectively. Under the PRIDE scheme, each patient may receive multiple doses, contribute multiple data points. Consequently, the total number of data points may exceed the number of patients, that is, $\sum_{k=1}^{K}n_{k}\geq n$.

In the CFO framework, the most appropriate dose level for an upcoming cohort is determined by competing the current dose against its two neighboring doses, similar to a two‐player game: One player tries to push the dose up, while the other tries to push it down, and once the equilibrium is reached, the MTD is identified. The determination of the dose level involves computing the odds of the true DLT rates $p_{k}$ being greater than the target DLT rate ϕ, defined as

$$O_{k}=\frac{\mathrm{Pr}\left(p_{k}>\phi |y_{k},n_{k}\right)}{\mathrm{Pr}\left(p_{k}\leq \phi |y_{k},n_{k}\right)},\;k=L,C,R,$$

where L,C,R correspond to the left, current and right doses. The reciprocal ${\bar{O}}_{k}=1/O_{k}$ represents the odds of $p_{k}\leq \phi$.

The odds $O_{C}$ reflects the evidence of the current dose being excessively toxic, and thus a large value of $O_{C}$ suggests dose de‐escalation. The odds ${\bar{O}}_{L}$ quantifies the evidence of left dose being overly tolerable, and a large value of ${\bar{O}}_{L}$ makes de‐escalation less favorable. Together, the odds ratio $O_{C}/{\bar{O}}_{L}$ serves as a measure of the tendency for de‐escalating to the left dose level, with a larger value of $O_{C}/{\bar{O}}_{L}$ supporting move towards the left (i.e., dose de‐escalation). Similarly, the odds ratio ${\bar{O}}_{C}/O_{R}$ measures the tendency to escalate to the right dose level (i.e., dose escalation). These odds ratios can be estimated using the posterior samples of $p_{k}$ from Section 2.1. Threshold values for the odds ratios, $\gamma_{L}$ and $\gamma_{R}$, can be pre‐determined by minimizing the probability of incorrect decisions. The decision rules of the CFO design are summarized in Table 1.

**TABLE 1:** Dose escalation and de‐escalation rules of the CFO design in searching for the MTD.

Right	Left	$O_{C}/{\bar{O}}_{L}>\gamma_{L}$	
		Yes (De‐ escalation)	No (Stay)
${\bar{O}}_{C}/O_{R}>\gamma_{R}$	Yes (Escalation)	Stay	Escalation
	No (Stay)	De‐escalation	Stay

To ensure the safety of retreated patients, we adopt the rules from Zhou et al. [17], by setting an upper limit of treatment cycles per patient to mitigate the risk of cumulative toxicity. Early stopping and dose elimination rules are also implemented to ensure safety at the trial level. Specifically, any dose level k identified as overly toxic, along with all higher dose levels, will be eliminated from the trial with no further dose assignment. If the lowest dose level is deemed overly toxic, as indicated by $\mathrm{Pr}\left(p_{1}>\phi ,y_{1}|n_{1}\right)>0.95$, the trial will be terminated.

The step‐by‐step procedure of the CFO design with the PRIDE scheme is detailed as follows:

1. Treat the first cohort of C patients at the initial dose level.
2. *Intra‐patient dose escalation*: If a patient has not experienced DLT and has not completed the prespecified treatment cycles, they are eligible for retreatment at higher dose levels.
3. *Dose level determination*: Under the PRIDE scheme, use the toxicity outcomes of enrolled patients to obtain posterior samples of the DLT rates across all doses. Calculate odds ratios and apply the decision rule in Table 1 to determine whether to escalate, de‐escalate, or remain at the current dose level.
4. *Cohort formation*: Assign a new cohort of patients to the selected dose. If fewer than C new patients are available, complete the cohort using eligible patients from the retreatment pool (patients treated earlier have higher priority to be selected due to a longer washout period).
5. Repeat steps 2–4 until the maximum sample size (N) is reached or a stopping rule is triggered (e.g., stop if the lowest dose is overly toxic), and finally select the MTD via the isotonic regression.

### 2.4 Incorporate the PRIDE Scheme to BOIN and CRM

The PRIDE scheme can be incorporated to other popular dose‐finding methods, such as BOIN and CRM. The BOIN design [6] determines dose escalation or de‐escalation based on comparison of the observed DLT rate at the current dose level with a pair of escalation ($\lambda_{e}$) and de‐escalation ($\lambda_{d}$) boundaries. Let ${\hat{p}}_{k}=y_{k}/n_{k}$, where $y_{k}$ and $n_{k}$ are the number of DLTs and the total number of patients treated at dose level k. The dose is escalated if ${\hat{p}}_{k}\leq \lambda_{e}$, de‐escalated if ${\hat{p}}_{k}>\lambda_{d}$, and remains unchanged otherwise. To integrate the PRIDE scheme into the BOIN design, we replace the estimated DLT rate ${\hat{p}}_{k}$ with the posterior estimate from PRIDE. These posterior estimates are then compared to the escalation and de‐escalation boundaries ($\lambda_{e}$, $\lambda_{d}$) to guide dose movement decisions. The PRIDE‐BOIN design improves efficiency through patient retreatment while maintaining the simplicity and robustness of BOIN.

The CRM [4] estimates the true DLT rate for dose k (i.e., $p_{k}$) using a logistic regression model,

$$\text{logit}\left(p_{k}\right)=\beta_{0}+\beta_{1}d_{k},$$

where $d_{k}$ is the effective dose of level k derived from the prior estimate (“skeleton”) of the toxicity probability [18, 22], and $\beta_{1}>0$ ensures monotonicity of DLT rates with doses. Using Bayesian updating, CRM obtains the posterior estimates of DLT rates and selects the dose whose toxicity probability is closest to the target rate ϕ as the MTD. Guo and Liu [18] proposed a similar principle to PRIDE by incorporating patient‐specific random effects $W_{i}∼𝒩\left(0,\sigma^{2}\right)$ into CRM. The toxicity probability model is expressed as

$$\text{logit}\left(p_{\mathrm{ik}}|d_{k},W_{i}\right)=\beta_{0}+\beta_{1}d_{k}+W_{i},$$

where $W_{i}$ captures individual variability. Incorporating the PRIDE scheme into BOIN and CRM illustrates its versatility and ability to facilitate various dose‐finding methods.

## 3 Simulations With Pride and Pride‐Fa Schemes

### 3.1 Simulation Setting

To evaluate the operating characteristics of the PRIDE and PRIDE‐FA schemes, we apply them to the CFO design and make a comparison with the AIDE and IPDE schemes. The main differences among these methods lie in their treatment of intra‐patient correlation and the strategy for retreating patients across treatment cycles. PRIDE and PRIDE‐FA model the intra‐patient correlation through random effects, whereas AIDE and IPDE do not account for the correlation. PRIDE and PRIDE‐FA differ in their retreatment strategy: PRIDE only allows retreatment to higher dose levels, whereas PRIDE‐FA permits retreatment to any dose level (except those already received by the same patient), including lower doses. AIDE and IPDE also restrict retreated patients to higher dose levels; however, they differ in the underlying safety principle guiding retreatment. AIDE prioritizes safety from a trial‐wide perspective, allowing retreatment to higher dose levels only if the trial itself escalates to those levels. IPDE considers safety from an individual perspective and immediately assigns a retreated patient to the next higher dose level, regardless of the trial movement.

To ensure patient safety and benefit, all simulations follow the rules for patient retreatment. Patients are eligible for retreatment if (i) they have not experienced DLT during the assessment period (cycle length), and (ii) they have not reached the maximum treatment cycles. Once the dose level for the next cohort has been determined, cohort slots are filled in the following priority: Patients who have been already enrolled in the trial but have not yet received any treatment are given the first priority (i.e., new patients are considered first). If additional slots remain, retreated patients are considered next, with preference given to those who have longer elapsed time since their last treatment, thereby maximizing the washout periods.

We conduct 5000 simulations under the trial settings in Table 2, considering both fixed and random scenarios. For PRIDE, PRIDE‐FA, AIDE, and IPDE, the maximum number of treatment cycles per patient is 3, that is, one patient can be treated up to three times (once as a new patient and up to twice as a retreated patient). For PRIDE and PRIDE‐FA, we take 2000 posterior samples for estimation and inference. We consider effective patients or effective sample size by including retreated patients. If one patient has been treated at 3 dose levels (first treated as a new patient and then twice as a retreated patient), then this subject would contribute 3 effective patients to the total sample size. The trial stops when either the total number of effective patients reaches 24 or when 12 effective patients have been accumulated at any dose level. Two sets of fixed scenarios (Sets A and B) are sourced from Jin and Yin [8] and Yuan et al. [23], as detailed in Table 3.

**TABLE 2:** Parameter setup in simulations.

Parameters	Values
Target DLT rate	0.33 or 0.2
Number of dose levels	5 or 7
Maximum effective patients limit	24
Per‐dose effective patients limit	12
Cohort size	3
Dose level assigned to the first cohort	1
Probability cutoff to eliminate overly toxic doses for safety	0.95
Probability threshold value for early stopping	0.95
Assessment window: weeks required to assess DLT	3
Inter‐patient arrival time: weeks between patients entering the trial	2

**TABLE 3:** The two sets of fixed scenarios considered for simulations with the target toxicity probability 0.33 in set A and 0.20 in set B as highlighted in boldface.

Scenarios	Dose levels						
	1	2	3	4	5	6	7
Set A (five‐dose scenarios)							
1	**0.33**	0.45	0.58	0.70	0.80		
2	0.18	**0.33**	0.52	0.60	0.70		
3	0.12	0.22	**0.33**	0.40	0.50		
4	0.01	0.02	0.03	**0.33**	0.50		
5	0.00	0.00	0.05	0.10	**0.33**		
6	0.45	0.55	0.65	0.75	0.85		
Set B (seven‐dose scenarios)							
1	0.05	**0.20**	0.46	0.50	0.60	0.70	0.80
2	0.02	0.05	**0.20**	0.28	0.34	0.40	0.44
3	0.01	0.05	0.10	**0.20**	0.32	0.50	0.70
4	0.01	0.04	0.07	0.10	0.50	0.70	0.90
5	0.01	0.05	0.10	0.14	**0.20**	0.26	0.34
6	0.01	0.02	0.03	0.05	**0.20**	0.40	0.50
7	0.01	0.04	0.07	0.10	0.15	**0.20**	0.25
8	0.01	0.02	0.03	0.04	0.05	**0.20**	0.45

To avoid cherry‐picking, we also generate random dose–toxicity scenarios using the method of Paoletti et al. [24], with the average probability difference around the target DLT rate (APDT) set to 0.05, 0.07, 0.1, and 0.15. We focus on the simulation results for Set B, while those for Set A are presented in Appendix A.

#### 3.1.1 Performance Metrics

We consider five designs for comparison: PRIDE, PRIDE‐FA, AIDE, IPDE in conjunction with CFO, and the standard CFO design. The evaluation focuses on accuracy, safety, and efficiency using the following metrics:

- Accuracy: This category evaluates the precision of MTD determination and patient allocation. It includes the percentage of correct MTD selection (MTD selection) and the percentage of patients allocated to the MTD (MTD allocation). Higher values within this category indicate greater accuracy.
- Safety: This category assesses safety and includes the percentage of selecting a dose above the MTD (overdose selection) and the percentage of patients allocated to dose levels above the MTD (overdose allocation). Lower values reflect improved safety and ethical considerations.
- Efficiency: This category measures sample size utilization and trial duration. It includes the percentage of sample size saved (sample size reduction), calculated as the relative difference in the number of patients using the standard CFO and the comparison design, and the average trial duration across simulations. A higher percentage of sample size saved and a shorter average trial duration indicate greater efficiency.

We follow the highest‐dose‐matter principle to count patients: A patient is counted as treated at the highest dose level among all doses they received. For example, if the MTD is dose level 2 and a patient receives dose levels 1, 2, and 3, this patient would be counted in the overdose allocation and excluded from MTD allocation.

### 3.2 Simulation Results

#### 3.2.1 Accuracy Evaluation

**FIGURE 1:**
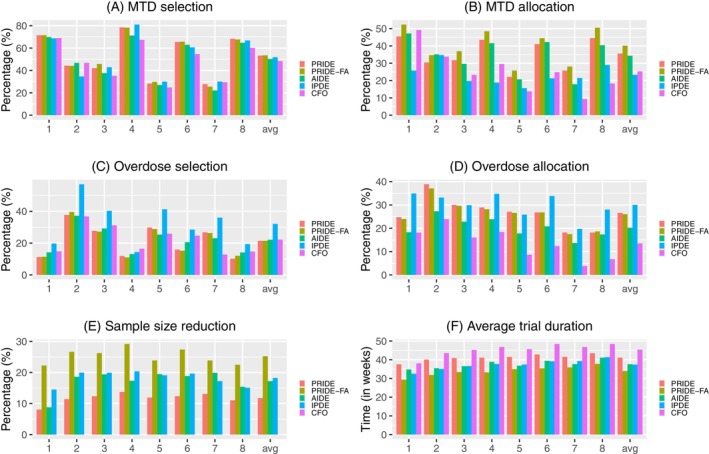
Simulation results of PRIDE, PRIDE‐FA, AIDE, IPDE and the CFO design with a target DLT rate 0.2 and seven dose levels under eight fixed scenarios. The numbers 1–8 on the x‐axis represent the eight scenarios, and “avg” represents the metrics averaged across these scenarios.

**FIGURE 2:**
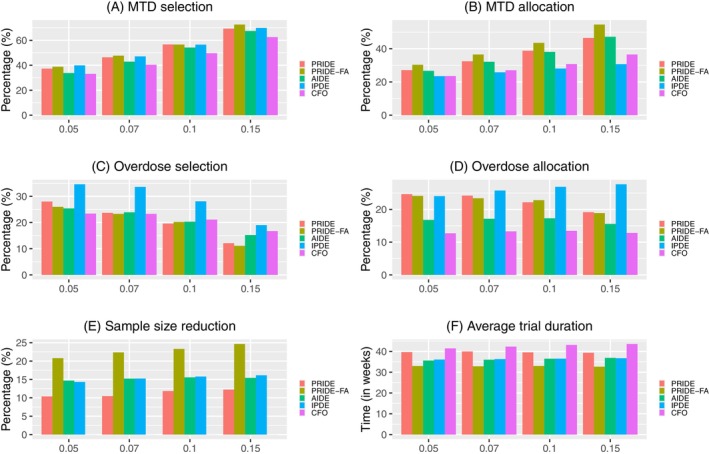
Simulation results of PRIDE, PRIDE‐FA, AIDE, IPDE and the CFO design with a target DLT rate 0.2 and seven dose level under random scenarios when the average probability difference around the target DLT rate varies from 0.05 to 0.15.

The results of PRIDE, PRIDE‐FA, AIDE, IPDE, and the standard CFO design are presented in panels A and B of Figure 1 (fixed scenarios) and Figure 2 (random scenarios). Due to its more efficient utilization of patient data through retreatment, CFO designs with intra‐patient dose escalation consistently outperform the standard CFO design. Among the four intra‐patient dosing designs, PRIDE‐FA yields the best performance in MTD selection, with PRIDE ranking second, followed by IPDE and AIDE. The advantage of PRIDE and PRIDE‐FA over AIDE is attributed to their consideration of the intra‐patient correlation using random effects. For MTD allocation, PRIDE‐FA again outperforms the others, followed by PRIDE and AIDE, with IPDE lagging significantly behind. The superior accuracy of PRIDE‐FA is ascribed to its appropriate modeling of the intra‐patient correlation and its strategic utilization of retreated patients. An increase in APDT makes the MTD more distinguishable and thus leads to improved accuracy in random scenarios. The results for a target DLT rate of 0.33 and five dose levels, provided in Appendix A, exhibit similar findings.

#### 3.2.2 Safety Evaluation

Based on panels C and D of Figures 1 and 2, IPDE yields the worst results in overdose selection and allocation for both fixed and random scenarios, reflecting its aggressive dose escalation strategy. Its dose escalation decisions are made solely based on the toxicity data of individual patients, disregarding safety data from other patients. In fixed scenarios, the overdose selection of PRIDE, PRIDE‐FA, AIDE, and CFO varies across different scenarios but shows little difference on average. In random scenarios, the overall safety improves as APDT increases.

#### 3.2.3 Efficiency Evaluation

The efficiency of the designs is evaluated using sample size reduction and average trial duration, as shown in panels E and F of Figures 1 and 2. For sample size reduction, PRIDE‐FA achieves the highest reduction rate compared to the other designs, reaching approximately 25%, whereas PRIDE shows the lowest reduction rate of around 10%–12%. AIDE and IPDE achieve an intermediate level reduction rate of around 15%. PRIDE‐FA leverages the flexible allocation for retreatment and assigns patients to any dose level, not solely to higher doses, which thereby can more effectively reduce the sample size required. The result of average trial durations aligns with that of the sample size reduction. PRIDE‐FA achieves the shortest trial duration, followed by AIDE, IPDE, and PRIDE, while CFO exhibits the longest trial duration. Similar results are observed for a target DLT rate of 0.33 in Appendix A.

#### 3.2.4 Overall Performance Comparison

PRIDE and PRIDE‐FA show the best performance in terms of accuracy while maintaining acceptable safety control. PRIDE‐FA achieves the highest sample size reduction and the shortest trial duration, indicating superior efficiency. AIDE performs moderately, with slightly lower accuracy in MTD selection than PRIDE and PRIDE‐FA, and inferior efficiency compared to PRIDE‐FA. IPDE's aggressive dose escalation strategy significantly compromises its safety.

### 3.3 Sensitivity Analyses

We conduct sensitivity analyses to assess the performance of PRIDE and PRIDE‐FA with respect to (i) the maximum sample size and (ii) prior distribution specifications.

#### 3.3.1 Sensitivity to Sample Size

We evaluate different maximum sample sizes N=9,12,15,24 on the operating characteristics of PRIDE and PRIDE‐FA. To isolate the effect of sample size, we remove the per‐dose effective patient stopping limit; the trial is only terminated when the total number of effective patients reaches the pre‐specified maximum sample size N. Results are summarized in Tables 4 and 5. As expected, increasing the sample size improves the MTD selection and leads to more patient allocation at the true MTD. For example, in Scenario 1 under PRIDE (Table 4), the MTD selection increases from 60.0% at N=9 to 63.7% at N=24, with more patients treated at the MTD. Similar trends are observed under PRIDE‐FA (Table 5), with the MTD selection rate rising from 58.4% at N=9 to 62.3% at N=24.

**TABLE 4:** True toxicity probability, and percentage of MTD selection and the average number of patients (shown in parentheses) treated at each dose level under the proposed PRIDE scheme when the maximum sample size (N) is 9, 12, 15, and 24.

Sample size	Dose level					None (%)
	1	2	3	4	5	
Scenario 1	**0.33**	0.45	0.58	0.70	0.80	
N=9	60.0 (2.88)	25.9 (3.27)	6.8 (1.28)	0.8 (0.24)	0 (0.02)	6.5
N=12	58.9 (4.13)	27.2 (4.32)	6.0 (1.68)	0.6 (0.34)	0 (0.02)	7.3
N=15	60.4 (5.60)	27.2 (5.37)	4.9 (2.01)	0.2 (0.41)	0 (0.03)	7.3
N=24	63.7 (10.83)	24.0 (7.86)	2.5 (2.50)	0.0 (0.43)	0 (0.04)	9.8
Scenario 2	0.18	**0.33**	0.52	0.60	0.70	
N=9	33.4 (1.25)	45.6 (3.44)	17.2 (2.49)	3.0 (0.72)	0.1 (0.09)	0.7
N=12	29.6 (1.63)	50.1 (4.80)	16.1 (3.42)	3.4 (0.98)	0.1 (0.15)	0.7
N=15	24.3 (2.12)	54.7 (6.20)	16.2 (4.17)	3.3 (1.24)	0.3 (0.29)	1.2
N=24	22.5 (3.91)	61.2 (11.14)	13.1 (5.91)	1.9 (1.55)	0.1 (0.36)	1.2
Scenario 3	0.11	0.22	**0.33**	0.40	0.50	
N=9	15.4 (0.66)	28.5 (1.99)	28.3 (2.68)	19.2 (1.92)	8.6 (1.06)	0.0
N=12	10.3 (0.76)	25.8 (2.29)	28.6 (3.51)	24.0 (2.90)	11.3 (1.80)	0.0
N=15	6.3 (0.78)	28.6 (3.01)	30.9 (4.42)	26.2 (3.87)	8.0 (2.10)	0.0
N=24	4.7 (1.18)	27.6 (4.99)	34.9 (7.37)	26.9 (6.51)	5.9 (3.04)	0.0
Scenario 4	0.01	0.02	0.03	**0.33**	0.50	
N=9	0.0 (0.03)	0.3 (0.07)	14.4 (0.78)	60.5 (4.50)	24.8 (3.59)	0.0
N=12	0.0 (0.03)	0.0 (0.06)	11.5 (0.73)	64.9 (6.19)	11.3 (4.77)	0.0
N=15	0.0 (0.03)	0.0 (0.06)	12.0 (0.94)	69.0 (7.91)	8.0 (5.51)	0.0
N=24	0.0 (0.03)	0.0 (0.06)	12.1 (1.74)	72.3 (13.70)	15.6 (7.63)	0.0
Scenario 5	0.00	0.00	0.05	0.10	**0.33**	
N=9	0.0 (0.00)	0.0 (0.00)	2.0 (0.28)	23.5 (3.30)	74.5 (5.42)	0.0
N=12	0.0 (0.00)	0.0 (0.00)	0.7 (0.20)	20.6 (3.69)	78.7 (8.07)	0.0
N=15	0.0 (0.00)	0.0 (0.00)	0.3 (0.20)	18.4 (2.84)	81.3 (10.78)	0.0
N=24	0.0 (0.00)	0.0 (0.00)	0.2 (0.21)	13.8 (3.44)	86.0 (18.95)	0.0
Scenario 6	0.45	0.55	0.65	0.75	0.85	
N=9	66.5 (3.98)	12.3 (2.70)	2.7 (0.60)	0.0 (0.09)	0.0 (0.00)	18.5
N=12	62.7 (5.63)	10.6 (3.34)	2.1 (0.73)	0.0 (0.10)	0.0 (0.00)	24.6
N=15	60.5 (7.40)	9.6 (3.73)	1.1 (0.78)	0.0 (0.12)	0.0 (0.00)	28.8
N=24	53.5 (12.63)	5.9 (4.53)	0.3 (0.84)	0.0 (0.12)	0.0 (0.00)	40.3

**TABLE 5:** True toxicity probability, and percentage of MTD selection and the average number of patients (shown in parentheses) treated at each dose level under the proposed PRIDE‐FA scheme when the maximum sample size (N) is 9, 12, 15, and 24.

Sample size	Dose level					None (%)
	1	2	3	4	5	
Scenario 1	**0.33**	0.45	0.58	0.70	0.80	
N=9	58.4 (3.01)	26.9 (3.34)	6.8 (1.32)	1.1 (0.26)	0 (0.03)	6.8
N=12	56.1 (4.34)	29.4 (4.46)	5.4 (1.69)	0.9 (0.32)	0 (0.05)	8.2
N=15	59.3 (5.76)	26.6 (5.27)	6.1 (2.08)	0.4 (0.51)	0 (0.04)	7.6
N=24	62.3 (10.77)	24.3 (7.91)	3.7 (2.64)	0.0 (0.53)	0 (0.04)	9.7
Scenario 2	0.18	**0.33**	0.52	0.60	0.70	
N=9	31.4 (1.30)	46.6 (3.45)	17.4 (2.58)	3.2 (0.74)	0.8 (0.12)	0.6
N=12	22.1 (1.69)	54.9 (4.86)	18.3 (3.56)	3.6 (0.98)	0.5 (0.20)	0.6
N=15	24.4 (2.21)	54.3 (6.00)	17.5 (4.36)	2.7 (1.29)	0.4 (0.25)	0.7
N=24	22.4 (3.72)	62.4 (11.21)	12.8 (6.14)	1.4 (1.63)	0.2 (0.31)	0.8
Scenario 3	0.11	0.22	**0.33**	0.40	0.50	
N=9	13.0 (0.67)	29.0 (1.93)	28.4 (2.63)	21.2 (2.14)	8.4 (1.10)	0.0
N=12	6.6 (0.78)	24.6 (2.27)	32.2 (3.39)	25.9 (3.16)	10.7 (1.81)	0.0
N=15	5.3 (0.79)	25.4 (2.75)	34.7 (4.67)	25.7 (3.89)	8.9 (2.19)	0.0
N=24	4.1 (1.06)	23.0 (4.31)	38.6 (7.89)	26.7 (6.46)	7.6 (3.42)	0.0
Scenario 4	0.01	0.02	0.03	**0.33**	0.50	
N=9	0.0 (0.03)	0.5 (0.09)	14.5 (0.83)	62.3 (4.52)	22.7 (3.50)	0.0
N=12	0.0 (0.03)	0.0 (0.07)	8.3 (0.72)	68.6 (6.37)	23.1 (4.71)	0.0
N=15	0.0 (0.03)	0.0 (0.06)	9.9 (0.85)	69.1 (7.81)	21.0 (5.61)	0.0
N=24	0.0 (0.03)	0.0 (0.06)	10.9 (1.57)	72.4 (13.61)	16.7 (7.89)	0.0
Scenario 5	0.00	0.00	0.05	0.10	**0.33**	
N=9	0.0 (0.00)	0.0 (0.00)	1.4 (0.30)	25.2 (3.33)	73.4 (5.37)	0.0
N=12	0.0 (0.00)	0.0 (0.00)	0.2 (0.22)	21.0 (3.68)	78.8 (8.07)	0.0
N=15	0.0 (0.00)	0.0 (0.00)	0.4 (0.20)	18.4 (2.90)	81.2 (10.74)	0.0
N=24	0.0 (0.00)	0.0 (0.00)	0.2 (0.21)	15.8 (3.74)	84.0 (18.67)	0.0
Scenario 6	0.45	0.55	0.65	0.75	0.85	
N=9	66.7 (4.34)	11.0 (2.63)	2.4 (0.57)	0.1 (0.08)	0.0 (0.01)	19.8
N=12	60.7 (6.12)	11.3 (3.19)	1.3 (0.68)	0.1 (0.08)	0.0 (0.01)	26.6
N=15	59.3 (7.53)	8.7 (3.77)	1.3 (0.84)	0.0 (0.12)	0.0 (0.01)	30.7
N=24	51.9 (12.61)	5.0 (4.47)	0.1 (0.94)	0.0 (0.11)	0.0 (0.01)	43.0

#### 3.3.2 Robustness to Prior Distributions

We examine the robustness of the PRIDE and PRIDE‐FA schemes under various prior distribution settings. The model includes fixed effects $\beta_{k}∼𝒩\left(\mu_{k},\sigma_{\beta }^{2}\right)$ with $\mu_{k}=\text{logit}\left(c_{k}\right)$ to ensure monotonicity, and subject‐specific random effects $W_{i}∼𝒩\left(0,\sigma^{2}\right)$, where $\sigma^{2}∼\mathrm{Inv}‐\text{Gamma}\left(\eta ,\eta \right)$. In PRIDE‐FA, a scaling parameter $\alpha_{2}∼𝒩\left(1,\sigma_{\alpha }^{2}\right)$ adjusts the influence of random effects. To assess the sensitivity to prior specifications, we vary the hyperparameters, including the fixed effect variance $\sigma_{\beta }^{2}$, the prior mean $c_{k}$, the parameter η for the inverse‐gamma prior on $\sigma^{2}$, and the normal distribution variance $\sigma_{\alpha }^{2}$ for $\alpha_{2}$. We also explore an alternative prior for $\sigma^{2}$ using a Half‐Cauchy prior on σ, σ∼Half‐Cauchy(0,τ). The full conditional distribution under the Half‐Cauchy prior for σ is

$$\log p\left(\sigma |\;W,D_{n}\right)\propto n\log\sigma +\frac{1}{2\sigma^{2}}\sum_{i=1}^{n}\;W_{i}^{2}+\log\left(\tau^{2}+\sigma^{2}\right).$$

Results are shown in Tables 6 and 7. Under most settings, the PRIDE design yields MTD selection ranging from 58% to 60%, while the PRIDE‐FA design maintains around 60% to 63%, suggesting both designs are robust to prior specifications.

**TABLE 6:** True toxicity probability, percentage of MTD selection and the average number of patients (shown in parentheses) treated at each dose level under the proposed PRIDE scheme with different prior distributions.

	Dose level					None (%)
	1	2	3	4	5	
Different $\sigma_{\beta }^{2}$ in $\beta_{k}∼𝒩\left(\mu_{k},\sigma_{\beta }^{2}\right)$						
Scenario 2	0.18	**0.33**	0.52	0.60	0.70	
$\sigma_{\beta }^{2}=1$	27.2 (1.33)	54.3 (7.30)	17.2 (6.54)	0.7 (1.18)	0.0 (0.06)	0.6
$\sigma_{\beta }^{2}=5$	23.5 (2.17)	58.4 (7.41)	16.1 (5.02)	1.3 (1.28)	0.1 (0.23)	0.6
$\sigma_{\beta }^{2}=10$	22.8 (2.27)	58.4 (7.50)	16.4 (4.77)	1.2 (1.28)	0.2 (0.23)	1.0
$\sigma_{\beta }^{2}=20$	21.8 (2.40)	59.9 (7.35)	15.5 (4.67)	1.6 (1.26)	0.2 (0.24)	1.0
Different $c_{k}$ in $\beta_{k}∼𝒩\left(\mu_{k},\sigma_{\beta }^{2}\right)$ with $\mu_{k}=\text{logit}\left(c_{k}\right)$						
Scenario 2	0.18	**0.33**	0.52	0.60	0.70	
$c_{k}=\left(0.05,\ldots ,0.25\right)$	22.1 (2.00)	58.1 (6.83)	16.9 (5.41)	2.1 (1.71)	0.2 (0.31)	0.6
$c_{k}=\left(0.1,\ldots ,0.5\right)$	22.8 (2.27)	58.4 (7.50)	16.4 (4.77)	1.2 (1.28)	0.2 (0.23)	1.0
$c_{k}=\left(0.15,\ldots ,0.75\right)$	22.9 (2.62)	59.9 (7.63)	14.6 (4.36)	1.2 (0.98)	0.2 (0.14)	1.2
Different η in $\sigma^{2}∼\mathrm{Inv}‐\text{Gamma}\left(\eta ,\eta \right)$						
Scenario 2	0.18	**0.33**	0.52	0.60	0.70	
η=0.5	23.3 (2.32)	58.0 (7.40)	16.7 (4.93)	1.4 (1.29)	0.0 (0.14)	0.6
η=1	22.8 (2.27)	58.4 (7.50)	16.4 (4.77)	1.2 (1.28)	0.2 (0.23)	1.0
η=2	22.1 (2.26)	58.6 (7.46)	17.4 (4.78)	1.0 (1.23)	0.1 (0.16)	0.8
η=5	24.1 (2.49)	57.7 (7.36)	15.6 (4.67)	1.5 (1.20)	0.0 (0.16)	1.1
Different τ in σ∼Half‐Cauchy(0,τ)						
Scenario 2	0.18	**0.33**	0.52	0.60	0.70	
τ=0.5	22.9 (2.06)	56.6 (7.20)	18.1 (5.10)	1.6 (1.37)	0.1 (0.16)	0.7
τ=1	22.6 (1.95)	55.8 (6.98)	19.4 (5.41)	1.3 (1.41)	0.2 (0.16)	0.7
τ=2	23.0 (1.86)	55.9 (7.03)	18.6 (5.42)	1.7 (1.39)	0.1 (0.15)	0.7
τ=5	23.1 (1.76)	56.9 (7.15)	17.8 (5.50)	1.4 (1.32)	0.2 (0.13)	0.6

**TABLE 7:** True toxicity probability at each dose, and percentage of MTD selection and the average number of patients (shown in parentheses) treated at each dose level under the proposed PRIDE‐FA scheme with different prior distribution.

	Dose level					None (%)
	1	2	3	4	5	
Different $\sigma_{\beta }^{2}$ in $\beta_{k}∼𝒩\left(\mu_{k},\sigma_{\beta }^{2}\right)$						
Scenario 2	0.18	**0.33**	0.52	0.60	0.70	
$\sigma_{\beta }^{2}=1$	26.6 (1.20)	55.0 (7.30)	16.5 (6.54)	1.3 (1.18)	0.0 (0.06)	0.6
$\sigma_{\beta }^{2}=5$	21.3 (1.68)	62.4 (7.03)	13.9 (4.77)	1.5 (1.14)	0.2 (0.19)	0.7
$\sigma_{\beta }^{2}=10$	21.6 (1.87)	62.9 (7.00)	13.1 (4.54)	1.7 (1.19)	0.1 (0.19)	0.6
$\sigma_{\beta }^{2}=20$	20.8 (1.92)	63.4 (6.93)	13.4 (4.51)	1.7 (1.19)	0.1 (0.21)	0.6
Different $c_{k}$ in $\beta_{k}∼𝒩\left(\mu_{k},\sigma_{\beta }^{2}\right)$ with $\mu_{k}=\text{logit}\left(c_{k}\right)$						
Scenario 2	0.18	**0.33**	0.52	0.60	0.70	
$c_{k}=\left(0.05,\ldots ,0.25\right)$	21.6 (1.62)	59.8 (6.44)	15.9 (5.17)	1.9 (1.52)	0.1 (0.25)	0.7
$c_{k}=\left(0.1,\ldots ,0.5\right)$	21.6 (1.87)	62.9 (7.00)	13.1 (4.54)	1.7 (1.19)	0.1 (0.19)	0.6
$c_{k}=\left(0.15,\ldots ,0.75\right)$	23.1 (2.12)	61.9 (7.20)	13.2 (4.17)	1.2 (0.94)	0.0 (0.09)	0.6
Different η in $\sigma^{2}∼\mathrm{Inv}‐\text{Gamma}\left(\eta ,\eta \right)$						
Scenario 2	0.18	**0.33**	0.52	0.60	0.70	
η=0.5	21.7 (1.70)	57.8 (6.68)	18.0 (4.90)	1.7 (1.38)	0.1 (0.18)	0.7
η=1	21.6 (1.87)	62.9 (7.00)	13.1 (4.54)	1.7 (1.19)	0.1 (0.19)	0.6
η=2	22.5 (2.03)	60.9 (6.76)	13.9 (4.48)	1.9 (1.34)	0.0 (0.20)	0.8
η=5	22.0 (2.03)	60.1 (6.79)	14.7 (4.46)	2.0 (1.25)	0.4 (0.21)	0.8
Different τ in σ∼Half‐Cauchy(0,τ)						
Scenario 2	0.18	**0.33**	0.52	0.60	0.70	
τ=0.5	21.7 (1.65)	57.5 (6.59)	17.9 (4.99)	2.3 (1.49)	0.0 (0.22)	0.6
τ=1	23.1 (1.67)	57.2 (6.55)	16.6 (5.10)	2.5 (1.42)	0.0 (0.22)	0.6
τ=2	21.9 (1.52)	57.3 (6.60)	17.7 (5.23)	2.4 (1.42)	0.1 (0.21)	0.6
τ=5	22.4 (1.44)	59.3 (6.65)	15.2 (5.32)	2.5 (1.40)	0.0 (0.20)	0.6
Different $\sigma_{\alpha }^{2}$ in $\alpha_{2}∼𝒩\left(1,\sigma_{\alpha }^{2}\right)$						
Scenario 2	0.18	**0.33**	0.52	0.60	0.70	
$\sigma_{\alpha }^{2}=0.25$	21.9 (1.86)	62.6 (7.03)	13.1 (4.54)	1.7 (1.18)	0.1 (0.19)	0.6
$\sigma_{\alpha }^{2}=1$	21.6 (1.87)	62.9 (7.00)	13.1 (4.54)	1.7 (1.19)	0.1 (0.19)	0.6
$\sigma_{\alpha }^{2}=10$	21.6 (1.83)	62.4 (7.00)	13.7 (4.58)	1.6 (1.17)	0.1 (0.19)	0.6
$\sigma_{\alpha }^{2}=20$	21.8 (1.84)	61.7 (6.96)	14.2 (4.61)	1.6 (1.19)	0.1 (0.19)	0.6

## 4 Trial Illustration

**FIGURE 3:**
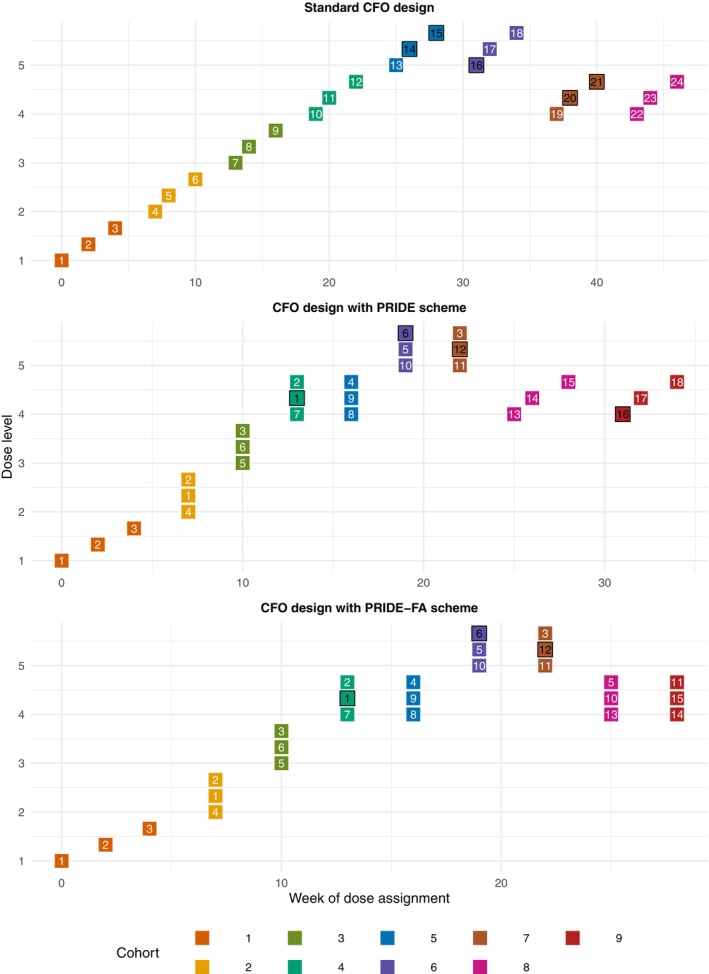
Trial illustration for PRIDE, PRIDE‐FA, and the CFO design. Each square represents a patient, with the number indicating the patient unique ID and the color indicating the same cohort members. A black‐bordered square with a black number denotes a DLT occurrence at that dose; a white number indicates no DLT.

For illustration, Figure 3 presents a phase I cancer trial conducted with the CFO design under PRIDE and PRIDE‐FA schemes, compared to the standard CFO design. The trial targets a DLT rate of 0.2 and evaluates seven dose levels with increasing DLT rates (0.01,0.05,0.10,0.20,0.32,0.50,0.70). Patients are enrolled every 2 weeks, and DLT assessment takes 3 weeks (i.e., the assessment window). Dose assignment for each new cohort is made only after all DLT outcomes from the previous cohort are available. The timing of patient enrollment is independent of the availability of DLT outcomes. For example, in the standard CFO, patient 4 is enrolled in week 6 but starts treatment in week 7 due to pending DLT results from patient 3. Each patient may receive up to three treatment cycles. The trial stops once 24 effective patients are accrued, or 12 effective patients have been treated at any dose level.

As depicted in Figure 3, under the standard CFO design, each patient is treated only once. The trial ends in week 49 after accruing 24 effective (different) patients. In contrast, PRIDE completes the trial in 37 weeks with only 18 different patients (contributing 24 effective data points to the trial). In this trial example, patients 1, 2, and 3, who constitute the first cohort and are enrolled on weeks 1, 2, and 4 respectively, do not experience DLTs and are thus eligible for retreatment. Their DLT outcomes become available by week 7, enabling a dose escalation decision. At that time, only one new patient, patient 4, is available. Patients 1 and 2, who have longer washout periods, are ready for retreatment to complete the cohort. This process continues till week 25, when dose level 4 is assigned to cohort 8. At this point, only patient 13 is newly enrolled, and no eligible retreated patients are available, requiring enrollment of additional patients. Patients 14 and 15 are enrolled in weeks 26 and 28 respectively to complete the cohort. Based on toxicity information of all enrolled patients, dose level 4 is assigned to cohort 9, bringing the total number of effective patients at this level to 12 and triggering completion of the trial. After a three‐week DLT assessment, the decision is to stay at dose level 4, and the trial concludes on week 37.

Although the dose movement pathways of PRIDE‐FA and PRIDE are identical, PRIDE‐FA achieves greater reduction in both sample size and trial duration. In the first seven cohorts, PRIDE‐FA uses the same patients as PRIDE. The difference arises starting from cohort 8, as PRIDE‐FA allows retreated patients to be assigned to any dose level (except repeating treatment at the same dose), rather than restricting them to only higher dose levels as in PRIDE. Patients 1, 2, and 3 reach the maximum number of treatment cycles and become ineligible. Among eligible patients, patients 4, 7, 8, and 9 have previously been treated at dose level 4. To preserve the property of PRIDE that observations at the same dose level must come from different patients, patients 5 and 10, who have not been treated at dose level 4 and have the longest washout periods, are assigned to cohort 8, along with newly enrolled patient 13. Due to the increased flexibility in dose assignment for retreated patients, PRIDE‐FA completes the trial with only 15 patients (still contributing 24 effective data points to the trial) and finishes 6 weeks earlier than PRIDE.

## 5 Discussion

The PRIDE and PRIDE‐FA schemes provide efficient, accurate, and ethical dose escalation for phase I clinical trials with small sample sizes. To minimize carryover effects, the design incorporates operational and modeling safeguards. Operationally, cohort slots are first assigned to newly enrolled patients. Retreated patients are considered next, with priority given to those with longer elapsed time since last treatment. This maximizes washout periods and minimizes residual effects from prior dose administrations before patients receive a new dose. Statistically, the inclusion of patient‐specific random effects captures intra‐patient correlations. In practice, investigators may further adjust the minimum washout time based on the pharmacokinetic and pharmacodynamic characteristics of the drug.

Although this study assumes binary toxicity outcomes (DLT or no DLT), PRIDE and PRIDE‐FA can be adapted to more complex trial settings, such as drug combination trials, multiple toxicity types, late‐onset toxicity, and phase I/II designs by jointly assessing toxicity and efficacy. These extensions can be realized by integrating PRIDE and PRIDE‐FA into existing designs tailored to these contexts. For instance, in trials with time‐to‐event late‐onset toxicity, they can be combined with the time‐to‐event CFO design proposed by Jin and Yin [19] or the fractional accumulative CFO design developed by Fang and Yin [20].

## Funding

The authors have nothing to report.

## Conflicts of Interest

The authors declare no conflicts of interest.

## Figures and Tables

**FIGURE A1:**
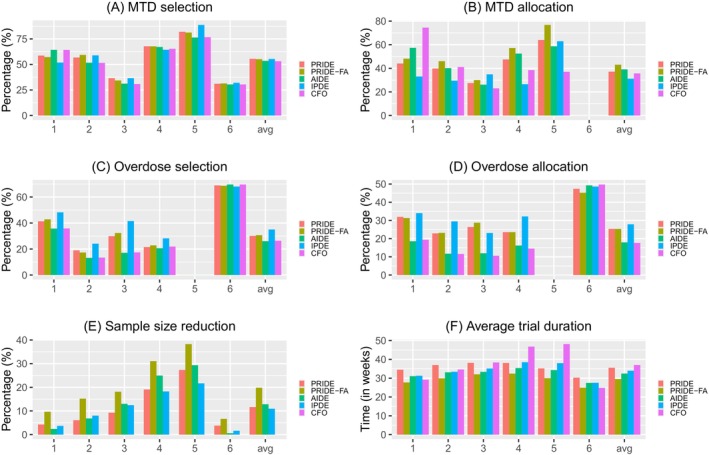
Simulation results of PRIDE, PRIDE‐FA, AIDE, IPDE and the CFO design with a target DLT rate 0.33 and five dose level under six fixed scenarios in Set A. The numbers 1–6 on the x‐axis represent the six scenarios, and “avg” represents the metrics averaged across the six scenarios.

**FIGURE A2:**
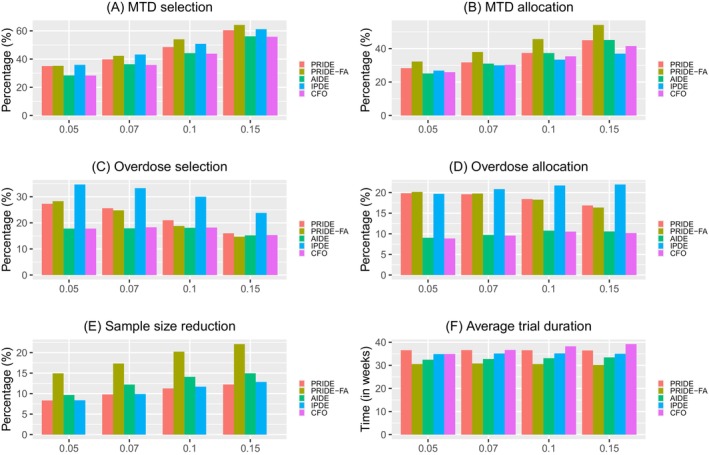
Simulation results of PRIDE, PRIDE‐FA, AIDE, IPDE and the CFO design with a target DLT rate 0.33 and five dose level under random scenarios when the average probability difference around the target DLT rate varies from 0.05 to 0.15.

## Data Availability

Data sharing not applicable to this article as no datasets were generated or analyzed during the current study.

## Appendix A More Simulation Results

We conduct numerical simulations for the six fixed scenarios in Set A of Table 3. These trial scenarios consider a target DLT rate ϕ=0.33 and five dose levels. The simulation results for the fixed and random scenarios using PRIDE, PRIDE‐FA, AIDE, IPDE, and the CFO design are shown in Figures A1 and A2, respectively.

In Scenario 6, all dose levels have true toxicity probabilities above the target DLT rate of 0.33, indicating that no dose is acceptable for selection as the MTD. The early stopping rule is thus triggered by recognizing that all doses are overly toxic and terminates the trial early, so as to protect patients from exposure to excessive toxicities. The thresholds for early stopping and dose elimination are both set to 0.95 by default. Lowering these thresholds would lead to less conservative strategies. We conduct numerical simulations for Scenario 6 with different thresholds 0.80,0.85,0.90,0.95. Figure A1 shows results under the PRIDE, PRIDE‐FA, AIDE, IPDE, and CFO designs, including the percentage of trials that stop early (None (%)), MTD selection, and the average number of patients treated at each dose.

When the threshold is 0.80, approximately 61% of trials stop early across all designs, thereby preventing patients from further exposure to unsafe doses. Under this conservative setting, only around 34% of trials select the lowest dose level (with a true DLT probability of 0.45) as the MTD. The average sample sizes are 8.52 (PRIDE), 7.96 (PRIDE‐FA), 8.18 (AIDE), 8.06 (IPDE), and 8.82 (CFO), reflecting the ability of all designs to detect excessive toxicity and terminate early when warranted. As the threshold increases to 0.95, the early stopping rate (None (%)) decreases to approximately 31%, while the proportion of trials that select the lowest dose level as the MTD increases to around 60%. Investigators can adjust the early stopping and dose elimination thresholds to control dose escalation: lower thresholds yield more conservative strategies, higher thresholds allow more aggressive escalation (Table A1).

**TABLE A1:** True toxicity probability, and percentage of MTD selection and the average number of patients (shown in parentheses) treated at each dose level under the PRIDE, PRIDE‐FA, AIDE, IPDE, and CFO designs under Scenario 6 with different early stopping thresholds 0.8, 0.85, 0.9, 0.95.

Threshold	Design	Dose level					None (%)	Sample size
		1	2	3	4	5		
0.80	Scenario 6	0.45	0.55	0.65	0.75	0.85		
	PRIDE	34.2 (5.63)	3.8 (2.32)	0.5 (0.52)	0 (0.05)	0 (0)	61.5	8.52
	PRIDE‐FA	34.3 (5.23)	3.5 (2.10)	0.5 (0.54)	0 (0.08)	0 (0.01)	61.7	7.96
	AIDE	35.3 (6.68)	3.5 (1.41)	0.1 (0.08)	0.1 (0.01)	0 (0)	61.0	8.18
	IPDE	32.7 (4.29)	4.6 (2.56)	0.8 (1.12)	0.1 (0.08)	0 (0.01)	61.8	8.06
	CFO	35.3 (7.30)	3.5 (1.42)	0.1 (0.09)	0.1 (0.01)	0 (0)	61.0	8.82
0.85	Scenario 6	0.45	0.55	0.65	0.75	0.85		
	PRIDE	42.4 (6.78)	5.1 (2.56)	0.7 (0.84)	0 (0.09)	0 (0)	51.8	10.28
	PRIDE‐FA	41.1 (6.17)	5.5 (2.32)	0.7 (0.79)	0 (0.10)	0 (0.01)	52.7	9.39
	AIDE	43.5 (8.33)	5.2 (1.79)	0.1 (0.10)	0.1 (0.02)	0 (0)	51.2	10.24
	IPDE	39.3 (5.07)	5.6 (3.54)	1.1 (1.43)	0.1 (0.13)	0 (0.01)	53.9	10.18
	CFO	43.5 (9.03)	5.2 (1.81)	0.1 (0.10)	0 (0.02)	0 (0)	51.2	10.97
0.90	Scenario 6	0.45	0.55	0.65	0.75	0.85		
	PRIDE	49.8 (6.81)	8.2 (2.54)	0.9 (0.86)	0 (0.12)	0 (0)	41.1	10.34
	PRIDE‐FA	47.3 (6.24)	8.3 (2.36)	0.7 (0.82)	0 (0.10)	0 (0.00)	43.7	9.53
	AIDE	51.2 (8.38)	6.1 (1.87)	0.1 (0.13)	0.1 (0.01)	0 (0)	42.5	10.38
	IPDE	45.6 (5.04)	7.8 (3.61)	1.4 (1.50)	0.2 (0.13)	0 (0.01)	45.0	10.29
	CFO	51.2 (9.10)	6.1 (1.90)	0.1 (0.13)	0.1 (0.01)	0 (0)	42.5	11.14
0.95	Scenario 6	0.45	0.55	0.65	0.75	0.85		
	PRIDE	59.2 (8.07)	8.4 (2.89)	1.3 (0.61)	0 (0.09)	0 (0)	31.1	11.66
	PRIDE‐FA	58.5 (7.64)	9.3 (2.68)	0.8 (0.56)	0 (0.09)	0 (0.01)	31.4	10.98
	AIDE	62.7 (9.24)	6.7 (1.82)	0.1 (0.11)	0.1 (0.02)	0 (0)	30.4	11.19
	IPDE	57.1 (5.72)	8.8 (3.74)	1.9 (1.65)	0.2 (0.13)	0 (0.01)	32.0	11.26
	CFO	62.7 (9.95)	6.7 (1.85)	0.1 (0.11)	0.1 (0.02)	0 (0)	30.4	11.94
